# Supplementary material for: Host JAK-STAT activity is a target of parasitoid wasp virulence strategies
Source: PLoS Pathog. 2024 Jul 1;20(7):e1012349. doi: 10.1371/journal.ppat.1012349 (PMC11244843; doi:10.1371/journal.ppat.1012349)
Supplement: S1 Table — Accession numbers for COI sequence data are given. (DOCX) [file ppat.1012349.s001.docx]

**Supplemental Table 1.** Species and strains names, and COI sequence accession numbers for all parasitoids used in this study.

| Species | Strain | Accession # |
| --- | --- | --- |
| *Leptopilina victoriae* | LvPhil | JQ808447 |
| *Leptopilina victoriae* | LvHaw | JQ808446 |
| *Leptopilina heterotoma* | Lh14 | JQ808444 |
| *Leptopilina heterotoma* | LhSw | JQ808445 |
| *Leptopilina guineaensis* | LgCam | JQ808442 |
| *Leptopilina guineaensis* | LgSA | JQ808443 |
| *Leptopilina maia* | LmAtl | JQ808440 |
| *Leptopilina clavipes* | LcNet | JQ808441 |
| *Leptopilina boulardi* | Lb17 | JQ808436 |
| *Leptopilina boulardi* | LbG486 | JQ808438 |
| *Ganaspis xanthopoda* | GxUg | JQ808434 |
| *Ganaspis xanthopoda* | GxHaw | JQ808433 |
