# Supplementary figures and images for: Host JAK-STAT activity is a target of parasitoid wasp virulence strategies
Source: PLoS Pathog. 2024 Jul 1;20(7):e1012349. doi: 10.1371/journal.ppat.1012349 (PMC11244843; doi:10.1371/journal.ppat.1012349)

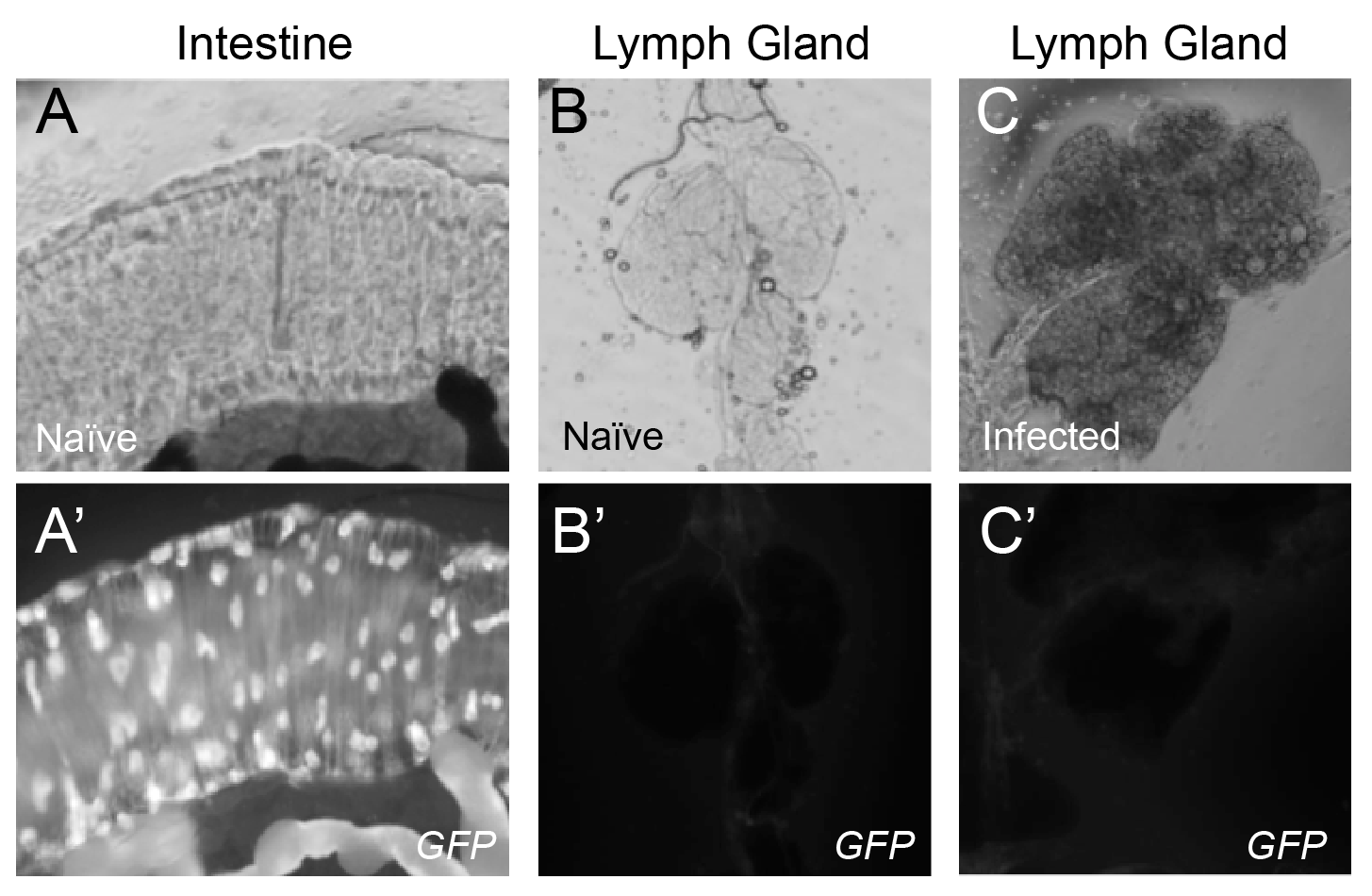

Supplement: S1 Fig — Brightfield (A-C) and fluorescence (A’-C’) images of 10xSTAT92E-GFP larvae. Strong fluorescence is seen in gut cells (A,A’). No fluorescence in seen in lymph gland dissected from naïve (B,B’) or LcNet infected (C,C’) larvae. (TIF) [file ppat.1012349.s002.tif]

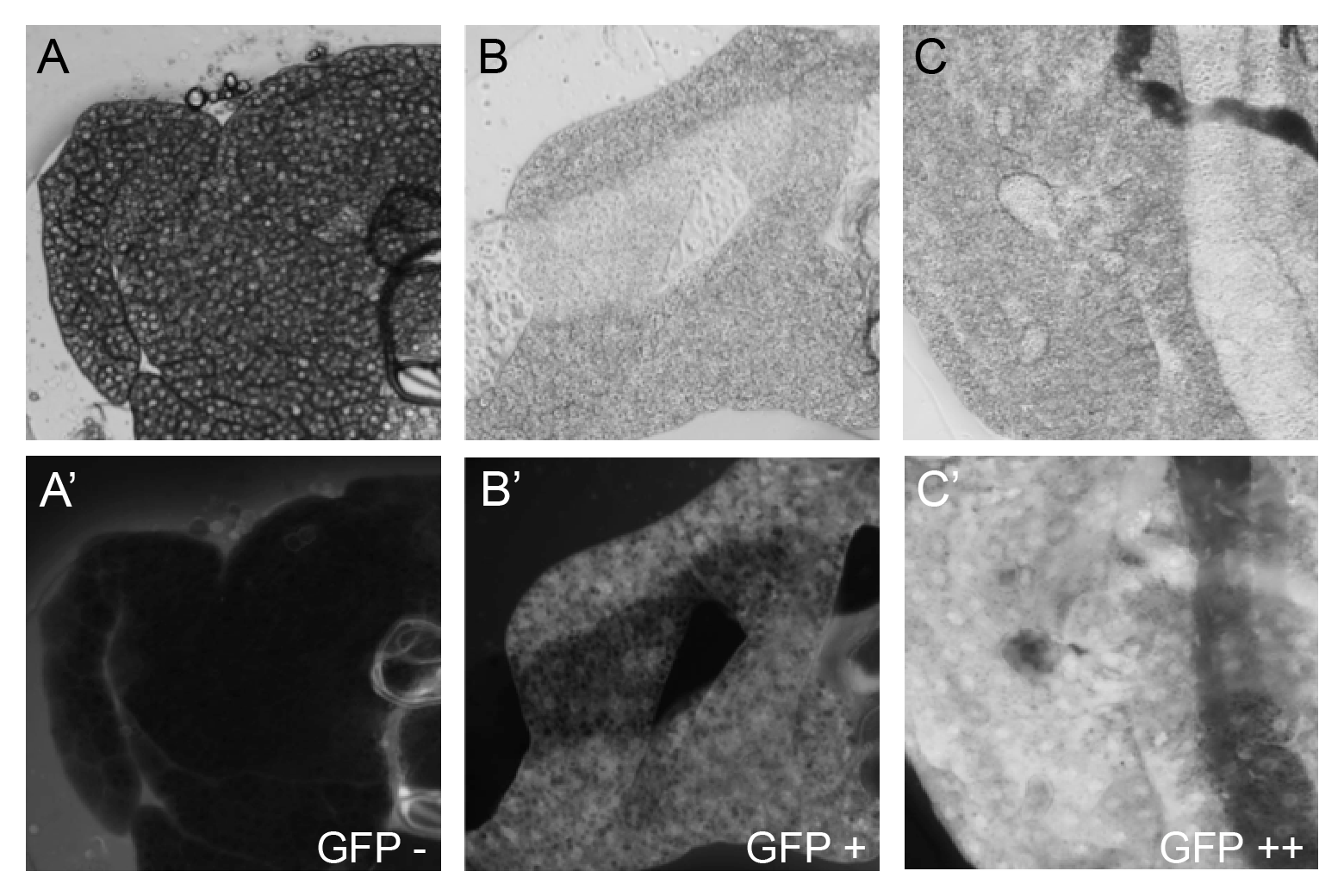

Supplement: S2 Fig — Brightfield (A-C) and fluorescence (A’-C’) images of fat bodies dissected from 10xSTAT92E-GFP larvae. Images are representative of the expression categories used in Table 1. A’ is representative of ‘-‘, B’ is representative of ‘+’ and C’ is representative of ‘++’. (TIF) [file ppat.1012349.s003.tif]

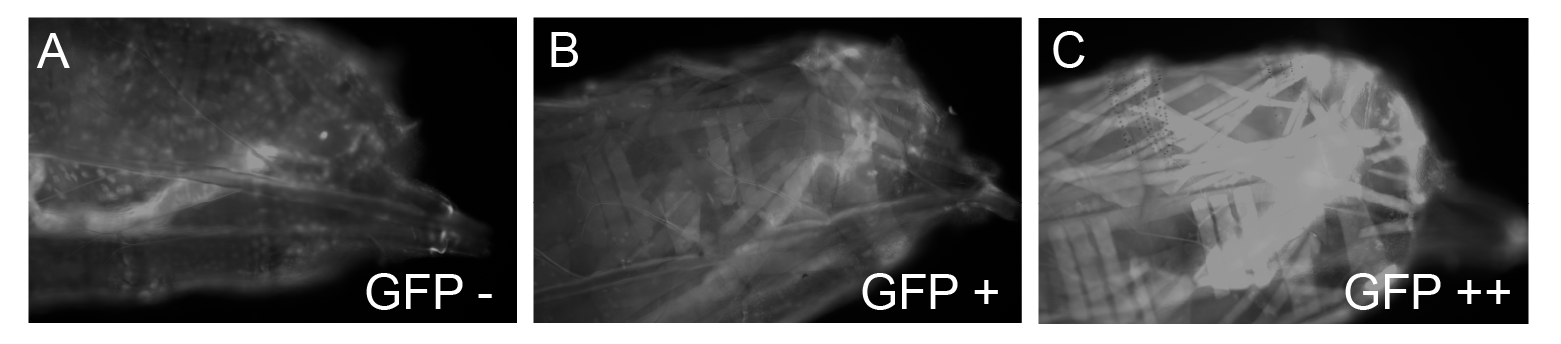

Supplement: S3 Fig — Images are representative of the expression categories used in Table 2. A is representative of ‘-‘, B is representative of ‘+’ and C is representative of ‘++’. All images were taken at the posterior end. (TIF) [file ppat.1012349.s004.tif]
